# Developmental telomere attrition predicts impulsive decision-making in adult starlings

**DOI:** 10.1098/rspb.2014.2140

**Published:** 2015-01-22

**Authors:** Melissa Bateson, Ben O. Brilot, Robert Gillespie, Pat Monaghan, Daniel Nettle

**Affiliations:** 1 Centre for Behaviour and Evolution, Institute of Neuroscience and Newcastle University Institute of Ageing, Newcastle University, Newcastle upon Tyne, UK; 2 Institute of Biodiversity, Animal Health and Comparative Medicine, University of Glasgow, Glasgow, UK

**Keywords:** impulsivity, self-control, telomere dynamics, state-dependent decision-making, body condition, European starling

## Abstract

Animals in a poor biological state face reduced life expectancy, and as a consequence should make decisions that prioritize immediate survival and reproduction over long-term benefits. We tested the prediction that if, as has been suggested, developmental telomere attrition is a biomarker of state and future life expectancy, then individuals who have undergone greater developmental telomere attrition should display greater choice impulsivity as adults. We measured impulsive decision-making in a cohort of European starlings (*Sturnus vulgaris*) in which we had previously manipulated developmental telomere attrition by cross-fostering sibling chicks into broods of different sizes. We show that as predicted by state-dependent optimality models, individuals who had sustained greater developmental telomere attrition and who had shorter current telomeres made more impulsive foraging decisions as adults, valuing smaller, sooner food rewards more highly than birds with less attrition and longer telomeres. Our findings shed light on the biological embedding of early adversity and support a functional explanation for its consequences that could be applicable to other species, including humans.

## 1. Introduction

One of the most important insights to emerge from behavioural ecology is that decisions should be state-dependent [1]. Animals in a poor biological state face reduced life expectancy, and as a consequence should make decisions that prioritize immediate survival and reproduction over long-term benefits [2]. Epidemiological studies in humans show that measures likely to be indicative of poor state are associated with altered time preferences. For example, low birth weight predicts both greater impulsivity [3,4] and accelerated reproduction [5,6]. However, attempts to demonstrate effects of state on time preferences in experimental animal models have met with mixed results [7]. We speculate that part of the reason for this inconsistency is that state, as defined by behavioural ecologists, is difficult to manipulate and measure. For example, an acute manipulation of food availability produces alterations in glycogen or fat reserves, but the effect that these changes have on probability of survival (which is what matters for evolutionary models of state-dependent decision-making) could be overshadowed by longer-term individual differences in anatomy, physiology and behaviour resulting from the quality of the developmental environment [8]. Thus, to test state-dependent models properly, we need a measure of state that integrates the effects of an animal's lifetime experience, and hence more accurately predicts life expectancy.

Telomeres are emerging as a plausible candidate to provide such a measure of state [9]. Telomeres are DNA ‘caps' found on eukaryotic chromosomes that shorten with age. Telomere loss is accelerated by various forms of stress exposure, with early-life stress being particularly damaging [10–12]. Furthermore, telomere length in humans and birds measured from blood prospectively predicts survival and/or health [13–18]. On the basis of these results, we hypothesize that telomere attrition is an integrative biomarker of biological state, and as such should be associated with the adaptive changes in decision-making predicted by state-dependent optimality models.

**Figure 1.**
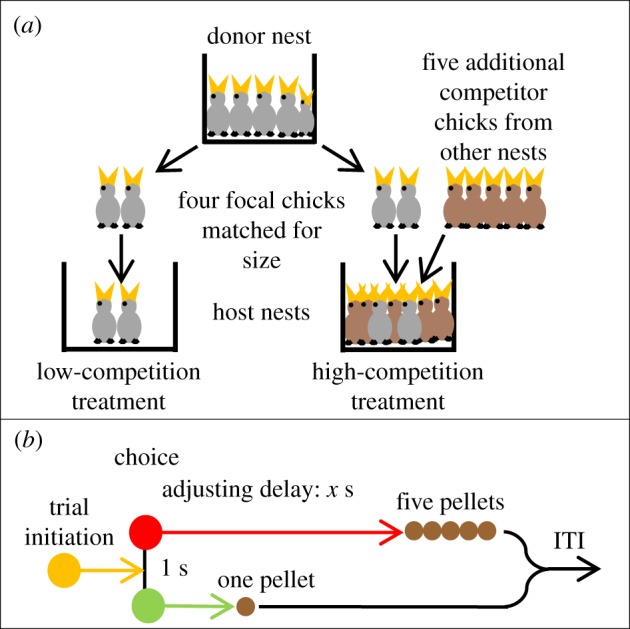
(*a*) Brood size manipulation. The diagram shows the creation of a single family of four focal chicks. A total of eight such families were created, yielding 32 focal chicks. (*b*) Inter-temporal choice task. The format of a single choice trial is shown. All trials began with an amber initiation light. One coloured key (here green) was assigned to the smaller sooner option (a 1 s delay to obtain one 45 mg pellet), and the other colour (here red) was assigned to the larger later option a longer, *x* s delay to obtain five 45 mg pellets). (Online version in colour.)

We tested this prediction in European starlings (*Sturnus vulgaris*), a long-lived, non-domesticated passerine bird species commonly used to test evolutionary and mechanistic models of decision-making [19]. We used a cohort of birds in which we had previously experimentally altered developmental telomere attrition via a brood size manipulation conducted on chicks in wild nests [20]. Briefly, pairs of focal siblings matched for weight were cross-fostered into nests where they faced either high or low competition for 12 days spanning the period during which most growth occurred (post-hatching day 3 to day 15, subsequently d3–d15; figure 1*a*), after which they were transferred to the laboratory for hand-rearing under uniform conditions. As we have shown elsewhere [20], this manipulation affected the birds' telomeres: the number of heavier competitors that a chick had on d15 predicted erythrocyte telomere attrition between d4 and d15. Furthermore, the effect was still evident at d55, after the birds had been reared under uniform laboratory conditions for 40 days [20]. Working on the assumption that developmental telomere attrition is a biomarker of state, we predicted that adult birds with greater developmental telomere attrition should be more impulsive, displaying a stronger preference for sooner food rewards when faced with a choice between ‘smaller sooner’ and ‘larger later’ rewards.

## 2. Material and methods

### (a) Study animals and husbandry

Subjects were 32 wild European starlings (*Sturnus vulgaris*) from a cohort of chicks hatched in the wild in May 2012 and subjected to a brood size manipulation described in detail elsewhere before being brought into the laboratory on d15 [20]. One chick failed to thrive and died before reaching independence, reducing the sample of birds available for behavioural testing to 31. Once the fledglings became independent (approx. four weeks post-hatch), they were transferred to two indoor aviaries (215 × 340 × 220 cm WDH; approx. 18°C; 40% humidity; 13 L : 11 D light cycle), provided with environmental enrichment and clean drinking water, and were fed ad libitum on domestic chick crumbs supplemented with dried insect food (Orlux insect paté), live mealworms and fruit.

Measurements of choice impulsivity took place when the birds were 6–14 months old and were fully grown. Replicates of eight birds (each comprising two genetic families) were caught from the aviary and moved to our operant laboratory (approx. 18°C; 40% humidity; 13 L : 11 D). Birds were housed in individual cages that served both for testing and as their home cages for the duration of testing. The cages measured 100 × 45 × 45 cm (WDH) and were identically furnished with two perches, a water bath and two water bottles. Each cage was additionally fitted with an operant panel permanently attached to one of the end walls comprising three horizontally aligned 4 cm-diameter pecking keys and one central food trough attached to a 45 mg pellet dispenser (see [21] for a full description).

While in individual cages the birds were food deprived overnight from 17.00 until testing began the following morning at 08.00. Water was always available ad libitum. Operant sessions lasted for a maximum of 5 h per day, and at 13.00 each day general husbandry was performed on the cages and the birds were given ad libitum food until 17.00. Each replicate remained in the operant laboratory for approximately six weeks, after which they were returned to the aviary and were replaced with the next two families. It took until the birds were approximately 14 months old (d428) to complete the testing of all eight families.

Birds were weighed on d55 and again when they were caught for transfer to individual cages and on return to the aviary. Tarsus length was measured on d55; the average of two independent measurements of both the right and left tarsus was used. As a measure of body condition, we derived residual body weight using the best-fitting regression equation for weight on d55 against tarsus length for all 31 birds (weight = 1.717 × tarsus + 15.437).

Soon after the end of the impulsivity experiments, the birds were permanently rehomed in a large outdoor aviary.

### (b) Telomere length and attrition measurements

Telomere lengths on d4, d15 and d55 for the birds used in the current paper were measured via quantitative PCR and have been published previously [20]. We took an additional blood sample at 14 months, after completion of the impulsivity experiment, and measured telomere length using identical methods. Owing to some failed assays, telomere length data were only available for 23 of the 31 birds. In this paper, we estimated telomere attrition over the developmental period (d4–d55) using the adjusted measure *D*, which is the difference in telomere length between d4 and d55, corrected for regression to the mean [22].

### (c) Operant training

Measurements of choice impulsivity began when the birds were 6–12 months old and were fully grown. Operant training procedures followed those outlined in [21]. First, the birds were auto-shaped to peck the centre amber key for a food reward. Once a bird started to peck the key, it progressed to a variable number of days of operant training. Each bird received daily sessions of 60 trials until it had pecked on at least 80% of trials in three sessions. When a bird had met this criterion it progressed to a generalization procedure to ensure operant responding when presented with the green and red key colours used in the impulsivity procedure (below). On successful completion of the generalization sessions birds progressed to the impulsivity procedure.

### (d) Impulsivity procedure

We used a standard inter-temporal choice task in which the birds made simultaneous choices between a smaller sooner food reward and a larger later food reward, titrating the value of the longer delay (*x*) at which individual birds became indifferent between the two options (figure 1*b*). To estimate indifference, we used an adjusting procedure [23]. Throughout the experiment, one colour (either green or red) was assigned to the smaller sooner (standard) option, and the other colour was assigned to the larger later (adjusting) option (colour assignment was constant within a bird but counterbalanced across birds and brood size treatments). In the smaller sooner option, there was always a 1 s delay to obtain one 45 mg pellet. In the larger later option, the *x* s delay varied from block to block of the experiment but the reward was always five 45 mg pellets delivered at a rate of 1 pellet s^−1^.

Each daily session comprised a maximum of 64 trials divided into 16 blocks of four trials. Sessions ended after 5 h if a bird had not completed 64 trials. Each block comprised two forced trials followed by two choice trials. At the start of each trial, the centre key was illuminated with amber light, and a single peck to this key was required to initiate the trial. On forced trials, following a response to the amber key, the amber light extinguished and either a red or green light appeared on the right or left key. A single peck to this light initiated the start of the programmed delay. Following the expiry of the programmed delay, a single further peck was required to extinguish the key light and initiate the delivery of reward. During reward delivery the hopper light was illuminated. Following the final pellet delivery the inter-trial interval (ITI) of 200 s began. Within each block, the two forced trials were chosen pseudo-randomly such that there was always one of each type (smaller sooner and larger later), with one being presented on each side. Choice trials were identical to forced trials with the exception that following the initiation peck, both side keys were illuminated (one in red and one in green). A single peck indicated the bird's choice and resulted in the non-chosen key being extinguished. In choice trials, the side on which each colour appeared was randomly chosen.

At the start of the experiment, the adjusting delay, *x*, was set to 1 s. At the end of each block, the *x* was updated according to the following rule: if the bird chose the standard option on both trials then the adjusting delay got 1 s shorter (*x* = *x* − 1); if the bird chose the adjusting option on both trials then the adjusting delay got 1 s longer (*x* = *x* + 1); and if the bird chose one of each option no change was made. The value of *x* had a minimum of 1 s but no maximum. The value of *x* at the end of each day was carried over to the start of the next day. Birds ran seven days a week and completed between 346 and 480 blocks (i.e. 1384–1920 trials).

### (e) Estimation of impulsivity

To estimate the indifference point—the value of *x* for which the two options were chosen equally often—we used the mean value of the adjusting delay, *x*, between the first block when a bird showed a preference for the larger later option and block 346 (the maximum block for which we had data from all birds; equivalent to 1384 trials). Means were based on a minimum of 243 blocks (i.e. 972 trials) per bird.

We expressed impulsivity in terms of *k*, a parameter that describes how rapidly the value of a given reward decreases as the delay to obtain it increases, where larger values of *k* equate to faster discounting of delayed rewards and hence greater choice impulsivity [23]. At indifference,2.1
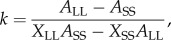
where *A*_SS_ and *A*_LL_ are the amount of reward in the smaller sooner and larger later options, respectively, and *X*_SS_ and *X*_LL_ are the delays in the same two options. We calculated values for *k* by substituting the following values in equation (2.1): *A*_LL_ = 5, *A*_SS_ = 1 (i.e. the numbers of pellets in the larger later and smaller sooner options, respectively), *X*_SS_ = 1 (i.e. the delay to reward in the smaller sooner option), and *X*_LL_ = the mean of the adjusting delay obtained from the adjusting procedure.

### (f) Statistics

Statistical analyses were conducted in R v. 3.0.1 using the package ‘nlme’. General linear mixed models (GLMMs) included random intercepts for genetic family to control for non-independence due to relatedness. The fixed effects included in each model are listed in the relevant results section. For all models, residuals were checked for normality and homogeneity of variance; where dependent variables required transformation to correct violation of assumptions, details are given in the results section. We used maximum-likelihood estimation throughout. Significance testing was carried out by the likelihood ratio test, which compares the change in deviance when a term is excluded from the model with the *χ*^2^ distribution with 1 d.f.

## 3. Results

### (a) Telomere dynamics

The analyses in this section are based on the subset of 20 birds for which we also had behavioural data (see below), but the results are qualitatively the same for the full set of 23 birds for which we had telomere data (statistics not shown); for completeness, the figures accompanying this section show the data from all 23 birds.

Telomere length at d4 was positively correlated with the difference in telomere length between d4 and d55 (Pearson correlation: *r*_18_ = 0.69, *p* < 0.001); birds that had longer telomeres at d4 suffered greater attrition. However, there was no significant correlation between telomere length at d4 and telomere attrition as measured by *D,* the difference in telomere length between d4 and d55 corrected for regression to the mean (Pearson correlation: *r*_18_ = −0.19, *p* = 0.4114). This pattern of results suggests that the former correlation could arise from measurement error [22]. We therefore used *D* as the measure of developmental telomere attrition in subsequent analyses to correct for this effect and to remove the need to control for telomere length at d4 in our models. A more negative value of *D* indicates greater attrition.

**Figure 2.**
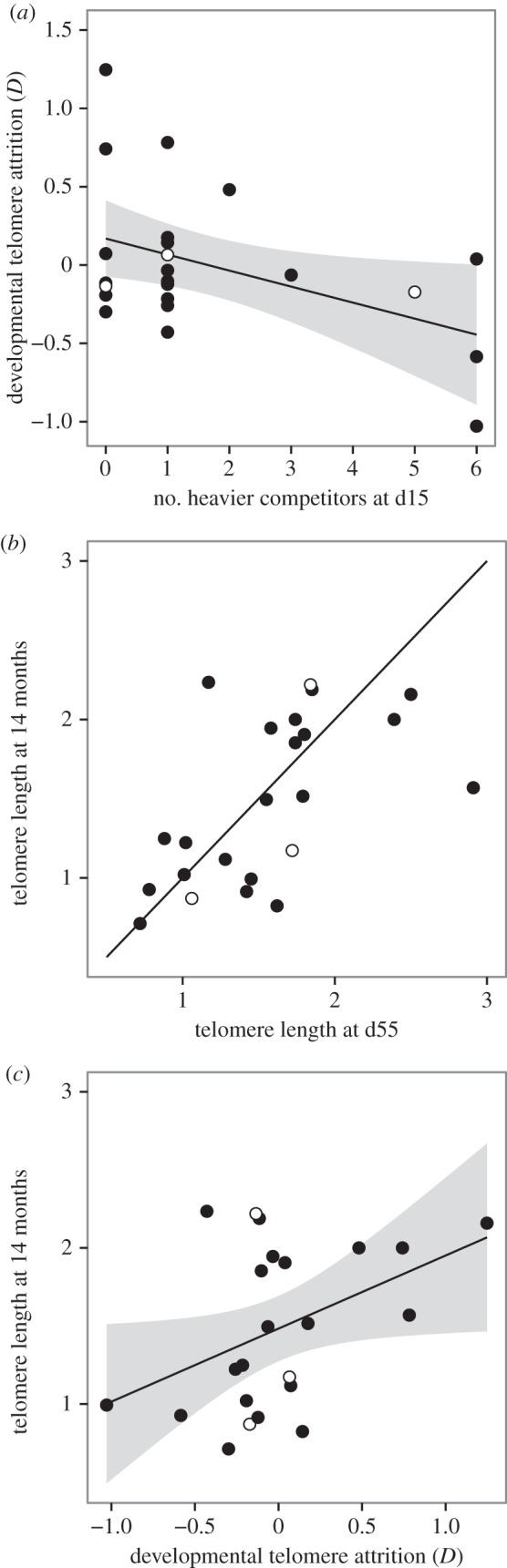
Telomere dynamics. (*a*) Having more, heavier competitors on d15 predicts greater developmental telomere attrition between d4 and d55. Telomere attrition is measured by *D* [22]; positive values of *D* indicate telomere lengthening over development and negative values indicate telomere loss. The solid black line is the line of best fit from a simple linear regression model, with 95% CIs shaded in grey. (*b*) Correlation between telomere length at d55 and telomere length at 14 months. The units of measurement are T/S ratios. The solid line shows the expectation if there was no change in telomere length. (*c*) Greater developmental telomere attrition (*D*) predicts shorter telomere length (T/S ratios) at 14 months. The graphs show data from all 23 birds for which we had telomere length measurements; the three birds lacking behavioural data are indicated with open circles.

To test whether developmental telomere attrition was predicted by our experimental manipulation, we fitted a model with ln(*D* + 2) as the dependent variable and the number of heavier competitors that a chick had at d15 as a continuous fixed predictor. The number of heavier competitors significantly predicted *D*, with birds with more heavier competitors experiencing greater telomere attrition (GLMM: 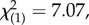 *p* = 0.0079; *B* ± s.e. = −0.07 ± 0.02; figure 2*a*).

Although telomeres did not shorten significantly between d55 and 14 months (paired *t*-test: *t*_19_ = −0.60 , *p* = 0.5554), some changes occurred (figure 2*b*). To explore whether the effects of the 12-day developmental manipulation lasted into adulthood, spanning the period of our behavioural measurements, we fitted a model with ln(telomere length at 14 months) as the dependent variable and developmental telomere attrition as a continuous fixed predictor. Developmental telomere attrition (*D*) significantly predicted telomere length at 14 months, with those birds with greater developmental attrition retaining shorter telomeres at 14 months (GLMM: 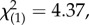 *p* = 0.0365; *B* ± s.e. = 0.31 ± 0.15; figure 2*c*).

### (b) Speed of discrimination learning

Three birds failed to complete the operant training for the impulsivity experiment and were excluded: one (low-competition treatment) developed diarrhoea and was removed from the experiment, one (low-competition treatment) refused to eat rodent pellets and one (high-competition treatment) was phobic of lit keys. Thus, we obtained behavioural data for 28 birds, of which 20 also had telomere data.

Since at the start of the impulsivity procedure the delay to reward in the two options was equal, we could use the point at which the birds started to show a preference for the larger later option as a measure of the speed at which they learnt that this option was associated with a larger reward. This is a cleaner measure of speed of learning than the number of trials taken to acquire the initial key-pecking response, because it is less likely to be confounded with neophobic responses to illuminated pecking keys [21]. A bird was defined as starting to show a preference for the larger later option when it first chose this option on 9/10 successive choice trials. We used the number of the first block in which this criterion was met as a measure of speed of learning. To test whether developmental telomere attrition predicted speed of learning, we fitted a model with speed of learning as the dependent variable and *D* as a continuous predictor. There was no significant effect of developmental telomere attrition (*D*) on the number of blocks taken to acquire the initial discrimination between the small and large options (GLMM: 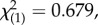 *p* = 0.4098; *B* ± s.e. = 10.48 ± 13.28).

### (c) Impulsivity

**Figure 3.**
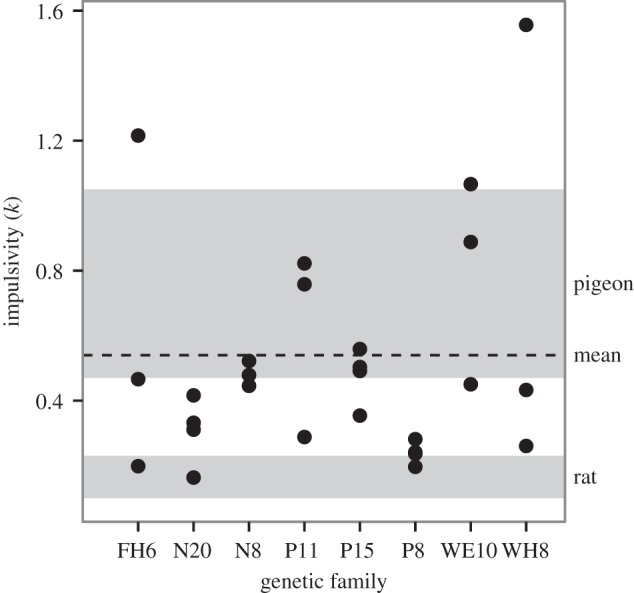
Estimates of choice impulsivity for individual starlings. Data are estimates of *k* for the 28 birds for which we obtained behavioural data. Birds are grouped by genetic family and the dashed line shows the mean value of *k* obtained. The shaded zones show the ranges of *k* reported in the literature for rats and pigeons.

The starlings had a mean value of *k* = 0.54 (s.d. = 0.35; *n* = 28), falling somewhere between values previously obtained for rats (less impulsive) and pigeons (more impulsive; figure 3). To examine the amount of variation in *k* explained by genetic family and by experimental replicate, we conducted a variance components analysis using maximum-likelihood estimation. The estimates of covariance parameters (±s.e.) were as follows: residual = 0.099 (±0.031), genetic family = 0.016 (±0.022), replicate = 0. Therefore, approximately 13.9% of the variance is explained by family and none by replicate. On this basis, we retained genetic family in our GLMMs as a random effect but ignored experimental replicate.

**Figure 4.**
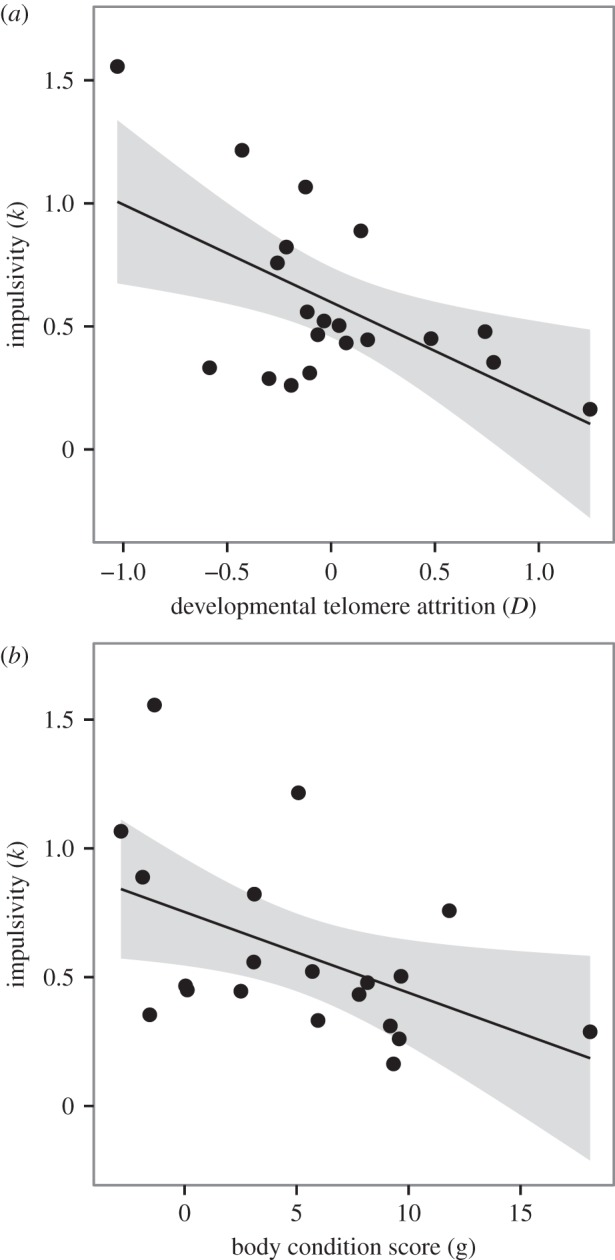
Predictors of impulsivity. (*a*) Greater developmental telomere attrition (*D*) predicts greater impulsivity. (*b*) Poorer body condition predicts greater impulsivity. In both panels, the data points represent the subset of 20 birds for which we obtained both developmental telomere lengths and estimates of impulsivity. The solid black line is the line of best fit from a simple linear regression model, with 95% CIs shaded in grey.

To test whether impulsivity was predicted by developmental telomere attrition, we fitted a model with ln(*k*) as the dependent variable, and developmental telomere attrition (*D*), body condition at the start of the impulsivity experiment and the interaction between these two factors as continuous fixed predictors (developmental telomere attrition and body condition were almost entirely uncorrelated: Pearson correlation, *r*_18_ < 0.01, *p* = 0.9980). Impulsivity (*k*) was significantly predicted by developmental telomere attrition (*D*), with greater impulsivity being associated with greater developmental telomere loss (GLMM: 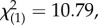 *p* = 0.0010; B ± s.e. = −0.29 ± 0.08; figure 4*a*). Impulsivity was also significantly predicted by body condition, with greater impulsivity being associated with a bird being relatively light for skeletal size at the start of the impulsivity measurements (GLMM: 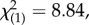 *p* = 0.0029; *B* ± s.e. = −0.23 ± 0.09; figure 4*b*). The interaction between telomere attrition and body condition explained no significant additional variation in impulsivity (GLMM: 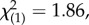 *p* = 0.1728, *B* ± s.e. = 0.15 ± 0.09). Note that if the raw differences in telomere length between d4 and d55 were used in place of *D* in the above model, the same pattern of results was obtained but, as would be expected, the effects were smaller.

Since developmental telomere attrition (*D*) was strongly correlated with telomere length at d55 (Pearson correlation, *r*_18_ = 0.75, *p* = 0.0001), considerations of collinearity precluded direct comparison of these two potential predictors of impulsivity in the same model. Re-running the above model with telomere length at d55 in place of *D* as a predictor showed that impulsivity (*k*) was also significantly predicted by telomere length at d55 (GLMM: 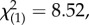 *p* = 0.0035; *B* ± s.e. = −0.31 ± 0.11); body condition also remained significant (GLMM: 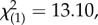 *p* < 0.0003; *B* ± s.e. = −0.04 ± 0.11) and the interaction between telomere length and condition not significant (GLMM: 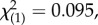 *p* = 0.7583, *B* ± s.e. = −0.02 ± 0.09).

Comparison of model fits showed that the model with telomere attrition had an Akaike information criterion (corrected for small sample size) value 4.03 units lower than the model with telomere length at d55. Calculation of the evidence ratio [24] suggested that model with telomere attrition is 7.52 times more likely to be the best-approximating model than the model with telomere length at d55.

## 4. Discussion

Our aim was to test the hypothesis that developmental telomere attrition is a measure of state, and hence should predict state-dependent decisions such as the relative value assigned to immediate versus delayed food rewards. Our results show that, as predicted, both developmental telomere attrition and absolute telomere length at independence predicted the impulsivity of foraging decisions in adult European starlings. Birds that had greater developmental telomere attrition between days 4 and 55 post-hatch, and birds that had shorter telomeres at day 55, had a stronger preference for smaller but more immediate food rewards than birds with less developmental attrition or longer telomeres.

Supporting some previous findings [25], we also found that impulsivity was significantly predicted by current body condition, with greater impulsivity being associated with a bird being relatively light for skeletal size at the start of the impulsivity measurements. Developmental telomere attrition and body condition were almost entirely uncorrelated, indicating that these were two independent measures of state. We suggest that telomere attrition is an integrative measure of the impact of developmental stress on biological state, whereas body condition is a more immediate measure of a single aspect of state, namely current energetic reserves. It might at first appear contradictory to our hypothesis that current body condition and telomere attrition should both independently predict impulsivity, because if telomere attrition is an integrative measure of state, as proposed, then it should embody current condition. However, since our telomere attrition measures were made at day 55, and our behavioural measures were in some cases made months later, it is possible that current body condition captures changes in state subsequent to the day 55 blood sample. In future studies, it would be interesting to acquire telomere length measures contemporaneously with behavioural measures to investigate how well current telomere length predicts decision-making. In our dataset, the parameter estimates (using both predictors scaled to make them comparable) indicate that the effect of telomere attrition on impulsivity was larger than the effect of current body condition (B ± s.e. = −0.29 ± 0.08 and −0.23 ± 0.09 respectively). This fits with substantial evidence that what happens during development can have profound and lasting effects on the adult behavioural phenotype. The importance of the developmental period in our birds is highlighted by the fact that developmental telomere attrition still significantly predicted telomere length over a year later at 14 months.

There is debate over whether telomere attrition or absolute telomere length is likely to be the best predictor of longevity [26], and whether loss or length is the best proxy for state. In this study, we are concerned with relatively young animals where the substantial cell senescence likely to be associated with short telomere length is unlikely to be a major factor in determining state. In our dataset, impulsivity is predicted better by developmental telomere attrition than by absolute telomere length at day 55, in agreement with what would be expected if telomere attrition is the best measure of state at this life-history stage. However, it is of considerable practical significance that telomere length (at day 55) is still a significant predictor of impulsive behaviour, since it suggests that even where longitudinal telomere measurements (and hence attrition values) are not available, variation in current telomere length could be used a reasonable proxy for variation in state.

Our results are consistent with the predictions of state-dependent models of decision-making in showing that individuals that are likely to be in a worse biological state, and hence with the lowest life expectancy, were also the most impulsive. Thus, we suggest that the individual variation in impulsivity that we have documented may represent adaptive responses of animals to their states. This is in contrast to the standard biomedical view that high choice impulsivity is pathological behaviour resulting from failure of top-down cognitive control [27,28]. Although it is possible to see our results as consistent with the biomedical view by arguing that both telomere attrition and adult impulsivity are independent measures of pathology, two pieces of evidence suggest that the adaptive interpretation deserves consideration. The first is that our brood size manipulation was within the normal range experienced by wild starlings, meaning that natural selection could reasonably have produced plastic behavioural strategies that allow chicks to respond adaptively to the impairment in state resulting from high sibling competition. Second, while telomere attrition predicted individual differences in impulsivity, it did not predict individual differences in speed of learning, which is a commonly used marker of basic cognitive performance [29]. This suggests that the birds were not generally cognitively impaired.

Our finding that what happens to a starling in the first two weeks of its life has a lasting effect on its telomeres, and that telomere attrition in turn predicts adult decision-making, raises questions about the biological embedding of early-life adversity and its effects on adult behaviour in other species. In humans, various kinds of adversity (including low birth weight and poverty) are strongly associated with impulsive decision-making [3,4,30], and it would be interesting to explore whether individuals with the most impulsive behaviour are also those with the shortest telomeres.

## Supplementary Material

Bateson_READ_ME.txt

## Supplementary Material

Bateson_impulsivity_data.csv

## Data accessibility

The data are available as the electronic supplementary material. R scripts for all of the analyses presented in this paper are available on request from the corresponding author.
